# Oxygen Extraction Based on Inspiratory and Expiratory Gas Analysis Identifies Ventilatory Inefficiency in Chronic Obstructive Pulmonary Disease

**DOI:** 10.3389/fphys.2021.703977

**Published:** 2021-07-28

**Authors:** Keisuke Miki, Kazuyuki Tsujino, Ryoji Maekura, Takanori Matsuki, Mari Miki, Hisako Hashimoto, Hiroyuki Kagawa, Takahiro Kawasaki, Tomoki Kuge, Hiroshi Kida

**Affiliations:** ^1^Department of Respiratory Medicine, National Hospital Organization Osaka Toneyama Medical Center, Toyonaka, Japan; ^2^Graduate School of Health Care Sciences, Jikei Institute, Osaka, Japan

**Keywords:** acidosis, carbon dioxide, cardiac function, exercise, gas exchange, ventilatory efficiency

## Abstract

**Aims:** In contrast to cardiovascular disease, low rather than high ventilatory inefficiency, evaluated by the minute ventilation-carbon dioxide output (*V'*_E_-*V'*_CO2_)-slope, has been recognized as being related to greater disease severity in chronic obstructive pulmonary disease (COPD). To better care for patients with cardiopulmonary disease, understanding the physiological correlation between ventilatory inefficiency and exercise limitation is necessary, but remains inadequate. Given that oxygen uptake (*V'*_O2_) evaluated by cardiopulmonary exercise testing (CPET) depends on both the ventilatory capability and oxygen extraction, i.e., the difference between inspiratory and expiratory oxygen concentration (ΔFO_2_), the aim of this study was to investigate the correlations between *V'*_E_-*V'*_CO2_-slope and the ΔFO_2_ during exercise and their physiological implications in patients with COPD.

**Methods:** A total of 156 COPD patients (mean age, 70.9 ± 7.2 years) with Global Initiative for Chronic Obstructive Lung Disease (GOLD) stages I–IV and 16 controls underwent CPET with blood gas analysis.

**Results:** With the progression of COPD, mechanical ventilatory constraints together with a slower respiratory frequency led to exertional respiratory acidosis. In GOLD IV cases, (1) decrease in the dependence of reduced peak *V'*_O2_ on *V'*_E_ led to an increase in its dependence on peak ΔFO_2_ during exercise; and (2) the ΔFO_2_-*V'*_CO2_-slope became steeper, correlating with the severity of exertional respiratory acidosis (r = 0.6359, *p* < 0.0001). No significant differences in peak exercise ΔFO_2_ or *V'*_E_-*V'*_CO2_-slope were observed among the various GOLD stages. In all subjects, including controls, peak exercise ΔFO_2_ had the strongest correlation with the *V'*_E_-*V'*_CO2_-slope (r = −0.8835, *p* < 0.0001) and correlated well with body mass index (r = 0.3871, *p* < 0.0001), although it did not correlate with the heart rate-*V'*_CO2_-relationship and *V'*_E_.

**Conclusions:** Ventilatory efficiency related to CO_2_ clearance might depend on exertional oxygen extraction in the body. Measuring ΔFO_2_ might be a key component for identifying ventilatory inefficiency and oxygen availability. Increasing ΔFO_2_ would help to improve ventilatory inefficiency and exercise tolerance separately from cardiac and ventilatory capability in COPD patients.

## Brief Summary

Understanding of the physiological correlation between ventilatory inefficiency and exercise limitation in chronic obstructive pulmonary disease (COPD) patients remains insufficient. The aim of this study was to investigate the correlation between ventilatory inefficiency and exertional variables and their physiological implications in COPD patients, using cardiopulmonary exercise testing and blood gas analysis. With the progression of COPD, the dependence of reduced peak oxygen uptake on the difference between inspiratory and expiratory oxygen concentration (ΔFO_2_) became relatively high, due to a decrease in its dependence on minute ventilation at peak exercise. Given that both O_2_ extraction capacity and ventilation affect exercise tolerance, increasing ΔFO_2_, as a measure of total oxygen availability in the body, might be the key to improving ventilatory inefficiency related to CO_2_ clearance, which, in turn, would lead to an increase in exercise tolerance based on the specific dysfunctions in COPD.

## Introduction

A better ventilatory response to exercise is helpful for improving exercise tolerance and exertional dyspnea in patients with cardiopulmonary diseases. Ventilatory inefficiency, indicated by a higher slope of the minute ventilation (*V'*_E_) vs. volume of exhaled carbon dioxide (*V'*_CO2_) relationship during exercise has been recognized as an index of greater disease severity and worse outcomes in cardiovascular diseases, such as heart failure (HF) and pulmonary arterial hypertension (PAH) (Wasserman et al., 1997; Sue, 2011; Dubé et al., 2016; Weatherald and Laveneziana, 2018; Nayor et al., 2020). In such diseases, excess ventilation is one of the most frequently recognized features to compensate for cardiovascular impairments, with the increase in respiratory frequency (*f*_R_), implying ventilatory inefficiency. In contrast, chronic obstructive pulmonary disease (COPD), in which cardiovascular impairment is not the primary feature, results in a higher ventilatory demand to compensate for the gas-exchange disorder, especially with the progression of the disease (Neder et al., 2017; Weatherald et al., 2018). Unexpectedly, however, it has been reported that not a high, but a low *V'*_E_-*V'*_CO2_-slope in COPD is associated with increasing COPD severity due to ventilatory abnormalities and mechanical constraints (Neder et al., 2015). To better care for patients with cardiopulmonary diseases, a deeper understanding of the physiological implication of the *V'*_E_-*V'*_CO2_ relationship and what is acceptable in common in the different scenarios may be needed.

In cardiopulmonary exercise testing (CPET), only gas flow and the concentrations of oxygen (O_2_) and carbon dioxide (CO_2_) are directly measured, with all other parameters being calculated using these measurements (Wasserman et al., 2012; Laviolette and Laveneziana, 2018). Peak oxygen uptake (*V'*_O2_) obtained from symptom-limited incremental CPET is an excellent indicator of exercise tolerance in cardiopulmonary diseases (Guazzi et al., 2012). Although *V'*_O2_ is calculated using the product of *V'*_E_ and the difference between inspiratory O_2_ concentration (FiO_2_) and expiratory O_2_ concentration (FeO_2_) (ΔFO_2_) (Wasserman et al., 2012; Laviolette and Laveneziana, 2018), little is known about the response of ΔFO_2_ to exertion and its relationship with ventilatory inefficiency.

The aim of this study, which included COPD patients across all Global Initiative for Chronic Obstructive Lung Disease (GOLD) stages and control subjects, was 1) to investigate whether the response of ΔFO_2_ as a measure of O_2_ extraction reflects the patterns of cardiopulmonary responses, including an evaluation of respiratory or metabolic acidosis, during incremental CPET together with blood gas analysis, and 2) to clarify the physiological implication of *V'*_E_ in relation to CO_2_ clearance, evaluated as the *V'*_E_-*V'*_CO2_-slope and the absolute values at the lowest *V'*_E_/*V'*_CO2_ during exercise, to investigate whether the *V'*_E_-*V'*_CO2_ relationship correlates with the ΔFO_2_ and clinical or cardiopulmonary variables.

## Methods

### Subjects

CPETs were performed in 4,557 patients with exertional dyspnea at our institution between May 1997 and August 2020. In this retrospective study, COPD patients and control subjects, who underwent CPET using a treadmill with an exertional evaluation of arterial blood gases and whose full data were stored, were selected. The diagnosis of COPD was confirmed based on the classification of the severity of airflow limitation in 2020 GOLD guidelines^1^. The exclusion criteria were patients who had absolute contraindications to clinical exercise testing (European Respiratory Society, 1997; Radtke et al., 2019), and those who had had a COPD exacerbation within the 2 months before CPET. Patients with comorbidities (e.g., severe cardiovascular disease, malignant tumors, active infection, asthma, pulmonary fibrosis, or neuromuscular disease) were also excluded. Age-matched control subjects with normal pulmonary function were recruited. No major cardiopulmonary diseases that could affect the results of CPETs were confirmed in the control subjects. This study included data from previous ethically-approved studies performed as screening for studies on COPD or control subjects at our institution. Thus, 156 patients with stable COPD and 16 control subjects were included in the study. All patients and control subjects provided written informed consent to undergo the protocols before the actual CPET. The institutional review board of the National Hospital Organization Osaka Toneyama Medical Center approved the study protocol, including the use of previous data sets (approval number: TNH-R-2020025), and the protocol was in accordance with the Declaration of Helsinki for experiments involving human subjects.

### Pulmonary Function Tests

Post-bronchodilator spirometry (CHESTAC 8800; CHEST M.I. Inc., Tokyo, Japan) was performed before exercise according to the recommendations of the American Thoracic Society (1995).

### Cardiopulmonary Exercise Testing (CPET)

**Figure 1:**
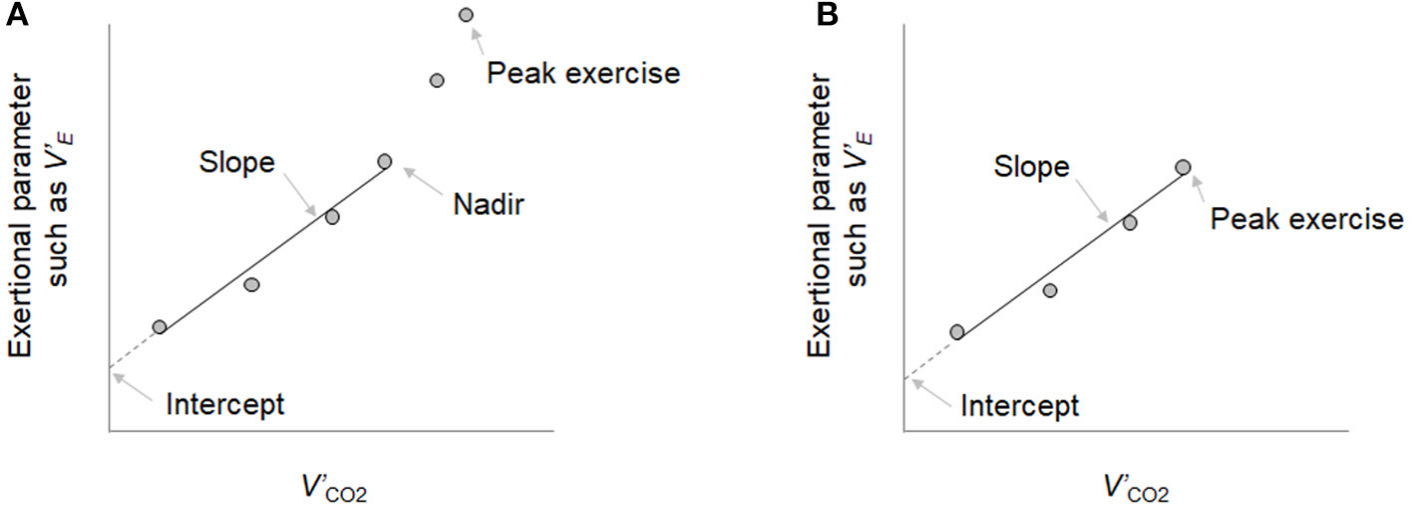
The exertional parameters-carbon dioxide output (*V'*co_2_) relationship. **(A)** When the respiratory compensation point (RCP) was observed during cardiopulmonary exercise testing, the minute ventilation (*V'*_E_)-*V'*co_2_-slope was calculated by linear regression, excluding the nonlinear part of the data after the onset of the RCP. **(B)** When no RCP could be identified, the *V'*_E_-*V*'_CO2_-slope was calculated from the data recorded from the start of exercise to the end of exercise. Similarly, the bicarbonate ion (HCO${}_{3}^{-}$)-*V*'_CO2_-slope, the ratio of inspiratory time to total respiratory cycle time (*T*i/*T*tot)-*V*'_CO2_ -slope, heart rate (HR)-*V*'_CO2_ -slope, and PaCO_2_-*V*'_CO2_-slope were determined in each parameter -*V*'_CO2_ plot. This is an original figure. No permission is required.

Symptom-limited incremental exercise tests were conducted on a treadmill using the Sheffield protocol or the two modified Sheffield protocols, as previously described (European Respiratory Society, 1997; Miki et al., 2009; Radtke et al., 2019). The exercise protocol that was likely to result in termination of the exercise test in about 8–10 min was selected based on the patient's daily activities and pulmonary function test results [especially the forced expiratory volume in 1 s (FEV_1_)]. Pre-exercise resting measurements were obtained during the steady-state period after at least 3 min of breathing through a mask. Exercise tests were performed without encouragement, especially during exercise to obtain reliable data, and were discontinued at subject exhaustion or signs indicating that exercise should stop. Gas exchange measurements were performed with the Aero monitor AE310S (Minato Medical Science Co., Ltd, Osaka, Japan): values of *V'*_E_, *V'*_O2_, *V'*_CO2_, *f*_R_, tidal volume (*V*_T_), the ratio of inspiratory time to total respiratory cycle time (*T*i/*T*tot), physiological dead space/tidal volume ratio (*V*_D_/*V*_T_), O_2_ pulse [*V'*_O2_/heart rate (HR)], ΔFO_2_, and end-tidal CO_2_ pressure (PetCO_2_) were measured breath-by-breath and collected as 30-s averages at rest, at 1 min and at 3-min intervals during exercise, and at the end of the exercise. Dyspnea intensity (10-point modified Borg category-ratio scale) and arterial blood samples for blood gas analyses, and plasma lactate assessments were collected at rest, during the last 15 s of the 1 min and at 3-min intervals, and at the end of the exercise, as previously described (Miki et al., 2009). CPETs were performed by three or four operators, and the data were analyzed by all the respiratory medicine house staff once a week. The *V'*_E_-*V'*co_2_-slope was calculated by linear regression, excluding the non-linear part of the data after the onset of the respiratory compensation point (Figure 1). When no respiratory compensation could be identified, the *V'*_E_-*V*'_CO2_-slope was calculated from the data recorded from the start to the end of the exercise. Similarly, the bicarbonate ion (HCO${}_{3}^{-}$)-*V*'_CO2_-slope, Ti/Ttot-*V*'_CO2_ -slope, HR-*V*'_CO2_ -slope, and PaCO_2_-*V*'_CO2_-slope were calculated; that is, the linear phase of each parameter–*V*'_CO2_ relationship was determined from each parameter -*V*'_CO2_ plot (each parameter during exercise was plotted on the Y-axis and the *V*'_CO2_ was plotted on the X-axis) (Figure 1). Positive and negative HCO${}_{3}^{-}$-*V*'_CO2_-slopes were defined as exertional respiratory acidosis and exertional metabolic acidosis patterns, respectively. The *V'*_E_-*V'*co_2_-intercept was defined as the nonzero point on the Y-axis, that is, *V'*_E_ (Figure 1). The *V'*_E_/*V*'_CO2_-nadir was defined as the lowest value during exercise. Positive and negative Ti*/*Ttot*-V'*_*CO*2_ *-*slopes were defined as non-prolonged expiration and prolonged expiration patterns, respectively. Predicted maximal voluntary ventilation (MVV) was calculated as FEV_1_× 35. Predicted maximum HR was calculated as 220–age in years (Wasserman et al., 2012).

### Statistical Analysis

Variables are expressed as means ± standard deviation (SD) unless otherwise stated. For continuous variables, (1) the Wilcoxon rank-sum test was used for comparisons between controls and patients of all GOLD stages, (2) the Kruskal–Wallis test was used for comparison among the groups consisting of the four GOLD stages and controls, followed by the Steel–Dwass test to carry out between-group comparisons, and (3) univariate analysis using Spearman's rank correlation coefficient was used to study the correlations between clinical variables. R squared was used to confirm how close the data were to the fitted regression line. The chi-squared test was used for categorical variables. A *p* < 0.05 was considered significant. Statistical analyses were performed using JMP software, version 11 (SAS Institute Inc., Cary, NC, USA).

## Results

A total of 156 patients distributed across all GOLD stages and 16 controls constituted the entire study sample (Table 1). All patients and controls were well-matched for age, sex, and body mass index (BMI), although BMI was lower in GOLD III and IV than in GOLD I groups.

**Table 1:** Baseline characteristics and resting pulmonary function of controls and COPD patients classified by the Global Initiative for Chronic Obstructive Lung Disease (GOLD).

**Subject (*n*)**	**Controls (16)**	**All COPD patients (156)**	**GOLD spirometric severity**			
			**I (11)**	**II (37)**	**III (66)**	**IV (42)**
Age, y	68.8 (10.3)	70.9 (7.2)	69.5 (6.2)	72.1 (8.1)	72.6 (6.6)	67.7 (6.5)^†c^
Sex, male/female (*n*)	13/3	142/14	11/0	33/4	58/8	40/2
BMI, kg · m^−2^	21.3 (2.9)	20.9 (3.3)	23.7 (1.7)	21.6 (3.2)	20.4 (3.6)^†a^	20.3 (3.0)^†a^
Pulmonary function test						
FEV_1_, L	2.48 (0.73)	1.13 (0.54)^*^	2.48 (0.34)	1.55 (0.30)^*^^†a^	0.97 (0.22)^*^^†a^†^*b*^	0.67 (0.12)^*^^†a^^†b^^†c^
%FEV_1_, % predicted	95.9 (19.2)	44.1 (19.4)^*^	91.6 (6.2)	61.2 (7.6)^*^^†a^	38.7 (5.7)^*^^†a^^†b^	24.9 (3.8)^*^^†a^^†b^^†c^
FEV_1_/FVC, %	80.5 (8.7)	45.1 (12.5)^*^	62.0 (4.7)^*^	54.3 (10.0)^*^	43.1 (8.4)^*^^†a^^†b^	34.7 (9.0)^*^^†a^^†b^^†c^
VC, L	3.34 (0.99)	2.87 (0.78)^*^	4.24 (0.75)	3.12 (0.68)^†a^	2.71 (0.66)^†a^^†b^	2.53 (0.59)^*^^†a^^†b^
%VC, %	107.2 (22.6)	91.5 (20.6)^*^	128.7 (15.6)	101.0 (15.9)^†a^	88.6 (16.2)^*^^†a^^†b^	78.0 (16.1)^*^^†a^^†b^^†c^
IC, L	2.28 (0.84)	1.78 (0.61)^*^	2.77 (0.74)	2.05 (0.43)^†a^	1.64 (0.56)^*^^†a^^†b^	1.51 (0.41)^*^^†a^^†b^
Medications (*n*)^‡^						
LAMA/LABA/ICS/SAMA/SABA/Theo	–	39/28/33/41/11/63	1/1/0/0/0/1	11/6/8/5/3/12	19/15/15/20/3/27	8/6/10/16/5/23
Primary comorbidities (*n*)						
Old myocardial infarction	–	5	1	0	2	2
Previous angina pectoris	–	9	1	1	4	3
Persistent atrial fibrillation	–	3	0	1	2	0
Lower extremity artery disease	–	3	0	1	1	1
Past tuberculosis disease	–	3	0	0	2	1

*Data are presented as means (standard deviation) unless otherwise stated. BMI: body mass index; FEV_1_: forced expiratory volume in 1 s; FVC, forced vital capacity; GOLD, Global Initiative for Chronic Obstructive Lung Disease; ICS, inhaled corticosteroid; LABA, long-acting β_2_-agonist; LAMA, long-acting muscarinic antagonist; SABA, short-acting β_2_-agonist; SAMA, short-acting muscarinic antagonist; Theo, theophylline; VC, vital capacity. Medications are presented separately. Pulmonary function test was performed after bronchodilator*.

* *p < 0.05 vs. controls;*

†a *p < 0.05 vs. GOLD I;*

†b *p < 0.05 vs. GOLD II;*

†c *p < 0.05 vs. GOLD III*.

‡ *Medication data were not obtained in four patients*.

### Pathophysiological Response and O_2_ Extraction During Exercise

Incremental exercise parameters at peak exercise in COPD patients and controls are shown in Table 2. From controls to GOLD IV, (1) the ratio of the prolonged expiration pattern (*p* < 0.0001) and the ratio of the respiratory acidosis pattern (*p* = 0.0025) increased during exercise, and (2) a slower *f*_R_ (*p* = 0.0004) and lower *V*_T_ (*p* < 0.0001), i.e., slow and shallow breathing, were confirmed, along with the lower Ti/Ttot, i.e., prolonged expiration (*p* < 0.0001), all of which led to the increased HCO${}_{3}^{-}$ level (*p* < 0.0001) with the higher arterial carbon dioxide tension (PaCO_2_) level (*p* < 0.0001) and the lower plasma lactate level (*p* < 0.0001), i.e., respiratory acidosis (Table 2). In GOLD IV, (1) although no significant differences in pH levels among GOLD stages were seen in this study population, PaCO_2_ was higher, and plasma lactate level was lower than in the other groups (Table 2); and (2) the peak *V'*_O2_ was lower than in the other groups (Table 2) and dependence of the reduced peak *V'*_O2_ on ΔFO_2_ became relatively high because of its decreased dependence on *V'*_E_ (Table 3). In addition, the ratio of ΔFO_2_ to *V'*_E_ at peak exercise was higher in the GOLD IV group than in the other groups (Table 2). Although there were no significant intrastage differences in peak exercise ΔFO_2_ and no differences between peak exercise ΔFO_2_ and resting ΔFO_2_ values among the GOLD stages, the ΔFO_2_-*V'*_CO2_-slope became steeper with the progression of the COPD stage (Table 2). The ΔFO_2_-*V'*_CO2_-slope correlated well with the PaCO_2_- *V'*_CO2_-slope, indicating exertional respiratory acidosis rather than the PaO_2_-*V'*_CO2_-slope, reflecting exertional hypoxemia (Table 4).

**Table 2:** Incremental exercise parameter of controls and COPD patients classified by the Global Initiative for Chronic Obstructive Lung Disease (GOLD).

		**GOLD spirometric severity**				
**Subject (*n*)**	**Controls (16)**	**I (11)**	**II (37)**	**III (66)**	**IV (42)**	***p*-value**
**At peak exercise**						
Dyspnea, Borg scale	7.1 (1.6)	5.8 (3.0)	7.1 (2.2)	6.8 (2.4)	6.8 (2.4)	0.7954
*V'*_O2_, mL · min^−1^ · kg^−1^	28.1 (4.6)	23.6 (4.2)	20.0 (4.8)^*^	16.8 (4.7)^*^^†a^^†b^	12.9 (3.4)^*^^†a^^†b^^†c^	<0.0001
R	1.12 (0.10)	1.08 (0.10)	1.03 (0.09)^*^	1.01 (0.10)^*^	0.93 (0.09)^*^^†a^^†b^^†c^	<0.0001
*V'*_E_, L · min^−1^	56.6 (12.3)	65.9 (18.4)	46.8 (12.3)^†a^	36.1 (9.1)^*^^†a^^†b^	26.5 (5.6)^*^^†a^^†b^^†c^	<0.0001
*V*_T_, mL	1,628 (485)	1,925 (412)	1,378 (303)^†a^	1,125 (309)^*^^†a^ ^†b^	944 (214)^*^^†a^^†b^^†c^	<0.0001
*f*_R_, breaths · min^−1^	37 (7)	35 (5)	35 (6)	33 (6)	29 (6)^*^^†b^^†c^	0.0004
Ti/Ttot	0.49 (0.03)	0.46 (0.03)	0.42 (0.04)^*^	0.38 (0.04)^*^^†a^^†b^	0.35 (0.04)^*^^†a^^†b^^†c^	<0.0001
*V'*_E_/ *V'*_O2_	36.1 (3.8)	43.1 (7.5)^*^	42.4 (8.6)	42.7 (10.3)^*^	39.8 (8.0)	0.0281
*V'*_E_/ *V'*_CO2_	32.5 (4.8)	40.0 (6.7)	41.6 (9.9)^*^	42.3 (9.5)^*^	42.9 (9.3)^*^	0.0005
*V*_D_/*V*_T_	0.21 (0.05)	0.22 (0.04)	0.27 (0.07)^*^	0.31 (0.07)^*^^†a^^†b^	0.35 (0.06)^*^^†a^^†b^^†c^	<0.0001
1- *V'*_E_/MVV, %	30.6 (13.5)	25.4 (11.8)	13.8 (16.4) ^*^	−7.4 (19.9)^*^^†a^^†b^	−15.4 (22.9)^*^^†a^^†b^	<0.0001
HR, beats · min^−1^	143 (14)	137 (17)	132 (21)	128 (14)^*^	120 (14)^*^^†a^^†c^	<0.0001
HR/predicted maximum HR, %	95.2 (8.3)	91.3 (11.1)	88.5 (13.8)	85.7 (9.3)^*^	79.5(9.3)^*^^†a^^†b^^†c^	<0.0001
O_2_ pulse, mL · beats^−1^	11.0 (2.0)	11.4 (3.2)	8.7 (2.7)^*^	6.9 (2.1)^*^^†a^ ^†b^	5.7 (1.3)^*^^†a^^†b^^†c^	<0.0001
ΔFO_2_, %	3.57 0.38)	2.96 (0.56)^*^	2.96 (0.58)^*^	2.95 (0.61)^*^	3.08 (0.60)^*^	0.0057
ΔFO_2_/*V'*_E_, % · L^−1^ · min	0.066 (0.015)	0.049 (0.020)	0.068 (0.024)	0.088 (0.033)^†a^ ^†b^	0.122 (0.037)^*^^†a^^†b^^†c^	<0.0001
pH	7.359 (0.031)	7.349 (0.032)	7.362 (0.038)	7.355 (0.039)	7.350 (0.033)	0.7082
PaO_2_, mmHg	82.8 (8.7)	65.3 (12.0)^*^	68.3 (14.1)^*^	61.8 (11.8)^*^	56.1 (9.6)^*^^†b^	<0.0001
PaCO_2_, mmHg	38.6 (3.3)	36.1 (3.9)	38.3 (5.7)	42.3 (6.5)^†a^ ^†b^	46.1 (5.8)^*^^†a^^†b^^†c^	<0.0001
PetCO_2_, mmHg	40.1 (5.6)	33.3 (5.5)	34.0 (6.3)^*^	35.9 (6.1)	38.3 (6.2)^†b^	0.0022
HCO${}_{3}^{-}$, mEq· L^−1^	21.5 (2.0)	19.8 (1.6)	21.4 (2.7)	23.2 (2.7)^†a^^†b^	25.2 (2.8)^*^^†a^^†b^^†c^	<0.0001
Plasma Lactate, mg · dL^−1^	41.0 (14.9)	43.7 (17.1)	35.1 (15.6)	28.7 (11.2)^*^^†a^	22.1 (9.0)^*^^†a^^†b^^†c^	<0.0001
**During exercise**						
Expiration pattern, prolonged/non-prolonged/ (n)	1/15	0/11	7/30	30/36	32/10	<0.0001
Exertional acidosis pattern, respiratory/metabolic (*n*)	1/15	1/10	7/32	21/45	19/23	0.0025
*V'*_E_-*V'*_CO2_-slope	28.0 (4.9)	35.2 (6.1)^*^	35.6 (8.9)^*^	35.5 (11.3)^*^	33.6 (10.9)	0.0081
*V'*_E_/ *V'*_CO2_-nadir	32.2 (4.9)	38.7 (6.0)^*^	41.4 (9.8)^*^	42.2 (9.4)^*^	42.4 (9.4)^*^	0.0003
*V'*_E_-*V'*_CO2_-intercept, L · min^−1^	6.7 (2.4)	5.8 (2.3)	6.4 (2.7)	6.2 (3.1)	6.2 (2.5)	0.9772
*V'*_E_/ *V'*_CO2_- rest minus nadir	27.0 (14.1)	20.9 (8.4)	20.3 (9.6)	20.3 (9.0)	19.6 (10.6)	0.2784
ΔFO_2_, % peak exercise minus rest	1.29 (0.62)	0.72 (0.52)	0.84 (0.52)	0.88 (0.50)	0.98 (0.52)	0.0505
ΔFO_2_-*V'*_CO2_-slope, % · L^−1^ · min	0.91 (0.39)	0.53 (0.47)	0.91 (0.50)	1.42 (1.05)^†a^ ^†b^	2.46 (0.19)^*^^†a^^†b^^†c^	<0.0001
HR-*V'*_CO2_-slope, beats · L^−1^	41.6 (9.0)	38.6 (12.8)	47.7 (22.5)	65.5 (31.6)^*^^†a^^†b^	77.6 (37.0)^*^^†a^^†b^	<0.0001

*Data are presented as means (standard deviation) unless otherwise stated. ΔFO_2_: difference between inspired oxygen concentration and expired oxygen concentration. Exertional metabolic acidosis pattern: negative HCO${}_{3}^{-}$-V'_CO2_ -slope; exertional respiratory acidosis pattern: positive HCO${}_{3}^{-}$-V'_CO2_ -slope (see the Methods for details); f_R_: breathing frequency; HR: heart rate; O_2_ pulse: V'_O2_/HR; PaCO_2_, arterial carbon dioxide tension; PaO_2_, arterial oxygen tension; PetCO_2_: partial pressure of end-tidal carbon dioxide, predicted maximum HR: 220–age (y); HCO${}_{3}^{-}$: bicarbonate ion; prolonged expiration pattern: negative Ti/Ttot-V'_CO2_-slope; non-prolonged expiration pattern: positive Ti/Ttot-V'_CO2_-slope (see the Methods for details); R: gas exchange ratio; Ti/Ttot: inspiratory duty cycle; V'_CO2_: carbon dioxide output; V_D_/V_T_: physiologic dead space/tidal volume ratio; V'_E_: minute ventilation; V'_E_/ V'_CO2_-slope, ΔFO_2_-V'_CO2_-slope, and HR-V'_CO2_-slope: the slope was determined by linear regression analysis of V'_E_, ΔFO_2_, or HR to V'_CO2_ obtained during exercise (see the Methods for details); V'_E_/ V'_CO2_-nadir: the lowest value during exercise (see the Methods for details); V'_O2_: oxygen uptake; V_T_: tidal volume*.

* *p < 0.05 vs. controls;*

†a *p < 0.05 vs. GOLD I;*

†b *p < 0.05 vs. GOLD II;*

†c *p < 0.05 vs. GOLD III. Estimated maximal voluntary ventilation (MVV) (L·min^−1^) was equal to forced expiratory volume in 1 s (FEV_1_) ×35*.

**Table 3:** Dominant correlations of the peak oxygen uptake classified by the Global Initiative for Chronic Obstructive Lung Disease (GOLD).

			**GOLD spirometric severity**							
**Subject (*n*)**	**Controls (16)**		**I (11)**		**II (37)**		**III (66)**		**IV (42)**	
	***r* ^**2**^**	***p*-value**	***r* ^**2**^**	***p*-value**	***r* ^**2**^**	***p*-value**	***r* ^**2**^**	***p*-value**	***r* ^**2**^**	***p*-value**
*V'*_E_, at peak exercise, L · min^−1^	0.62	0.0003	0.56	0.0082	0.52	<0.0001	0.57	<0.0001	0.39	<0.0001
ΔFO_2_, at peak exercise, %	0.15	0.1556	0.28	0.0947	0.35	0.0001	0.34	<0.0001	0.29	<0.0001

*ΔFO_2_, difference between inspired oxygen concentration and expired oxygen concentration; V'_E_, minute ventilation*.

**Table 4:** Correlations of ΔFO_2_-*V'*_CO2_-slope classified by the Global Initiative for Chronic Obstructive Lung Disease (GOLD).

			**GOLD spirometric severity**							
**Subject (*n*)**	**Controls (16)**		**I (11)**		**II (37)**		**III (66)**		**IV (42)**	
	***r***	***p*-value**	***r***	***p*-value**	***r***	***p*-value**	***r***	***p*-value**	***r***	***p*-value**
PaO_2_-*V'*_CO2_-slope, mmHg · L^−1^ · min	−0.1480	0.5987	0.3158	0.3441	0.0244	0.8861	−0.1617	0.1946	−0.2137	0.1742
PaCO_2_-*V'*_CO2_-slope, mmHg · L^−1^ · min	0.3220	0.2419	0.3586	0.2788	0.1903	0.2593	0.4646	<0.0001	0.6359	<0.0001
Lactate-*V'*_CO2_-slope, mg · dL^−1^ · L^−1^ · min	−0.0448	0.8740	−0.5904	0.0558	−0.1745	0.3015	−0.2724	0.0269	−0.0318	0.8418
pH-*V'*_CO2_-slope, mmHg · L^−1^ · min	−0.1289	0.6471	0.2171	0.5214	−0.1407	0.4061	−0.2482	0.0445	−0.3470	0.0243
*f*_R_-*V'*_CO2_-slope, breaths · L^−1^	−0.1505	0.5923	−0.3945	0.2299	−0.2223	0.1860	0.0324	0.7961	−0.3524	0.0221

*ΔFO_2_, difference between inspired oxygen concentration and expired oxygen concentration; f_R_, breathing frequency; PaCO_2_, arterial carbon dioxide tension; PaO_2_, arterial oxygen tension; V'_CO2_, carbon dioxide output; V'_E_, minute ventilation. ΔFO_2_-V'_CO2_-slope, PaO_2_-V'_CO2_-slope, PaCO_2_-V'_CO2_-slope, lactate-V'_CO2_-slope, pH-V'_CO2_-slope, or f_R_-V'_CO2_-slope were determined by linear regression analysis of ΔFO_2_, PaO_2_, PaCO_2_, plasma lactate level, pH, or f_R_ to V'_CO2_ obtained during exercise (see the Methods for details)*.

### Correlations Between Ventilatory Inefficiency and the Assessed Parameters

**Figure 2:**
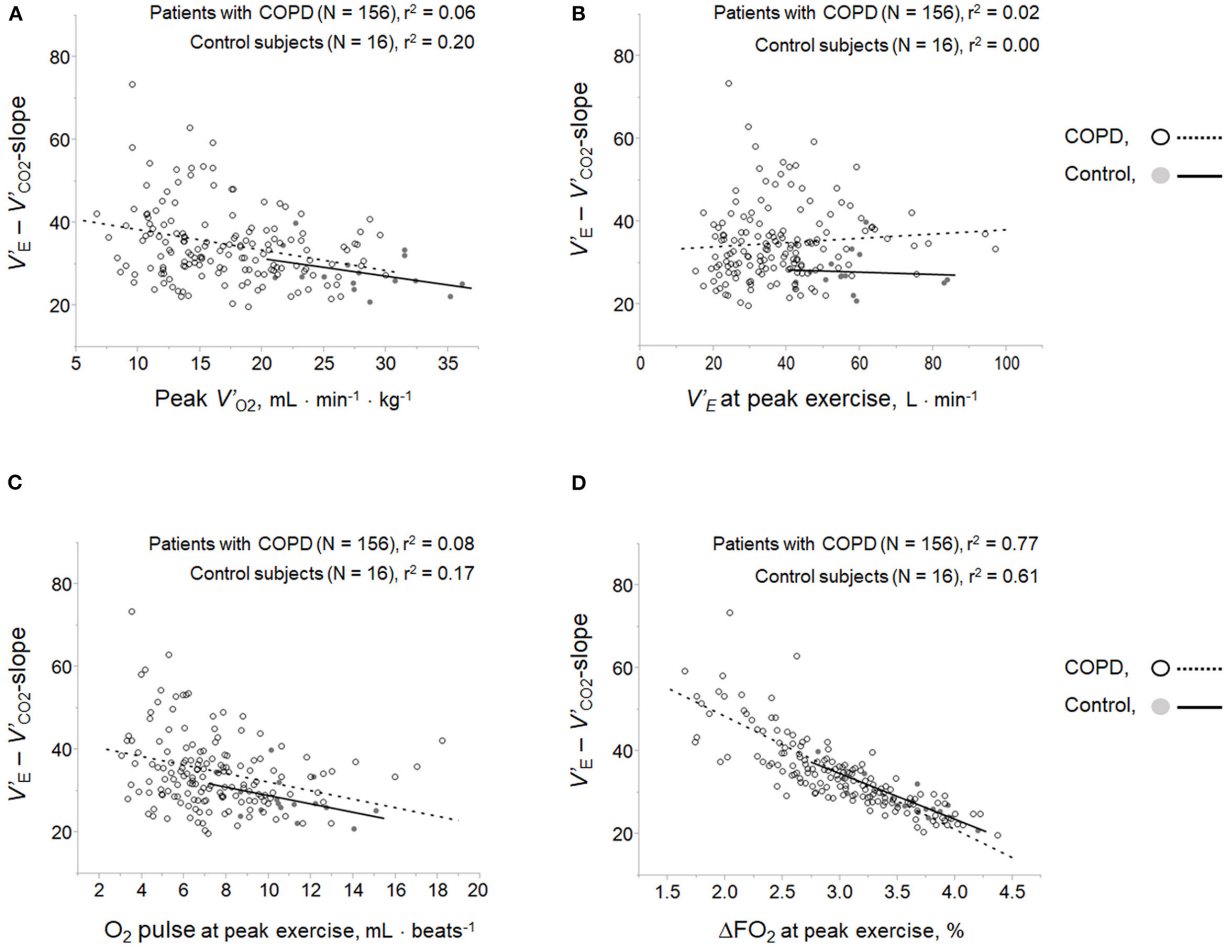
Correlations of ventilatory inefficiency with **(A)** peak *V'*_O2_, **(B)** *V'*_E_ at peak exercise, **(C)** O_2_ pulse at peak exercise, and **(D)** ΔFO_2_ at peak exercise. ΔFO_2_, difference between inspired and expired oxygen concentration; O_2_ pulse, *V'*_O2_/heart rate; *V'*_E_, minute ventilation; *V'*_O2_, oxygen uptake.

Respiratory compensation points during CPET were observed in 9/16 (56%) controls, 6/11 (55%) GOLD I cases, 11/37 (30%) GOLD II cases, 11/66 (17%) GOLD III cases, and 9/42 (21%) GOLD IV cases. No significant intrastage differences were confirmed in *V'*_E_/ *V'*_CO2_ at peak exercise, *V'*_E_-*V'*_CO2_-slope, the *V'*_E_/ *V'*_CO2_-nadir, *V'*_E_-*V'*_CO2_-intercept, and the difference between the *V'*_E_/ *V'*_CO2_-nadir and *V'*_E_/ *V'*_CO2_ at rest among the four GOLD stages, although the *V*_D_/*V*_T_ at peak exercise was higher in GOLD stage III and IV patients than at the milder stages and in controls (Table 2). As shown in Figures 2A–D, in both COPD patients and controls, the *V'*_E_-*V'*_CO2_-slope correlated strongly with the peak exercise ΔFO_2_ (oxygen extraction), compared with peak *V'*_O2_ (exercise tolerance), *V'*_E_ (ventilatory capability), and O_2_-pulse (cardiac function). Correlations of the *V'*_E_-*V'*_CO2_-slope with the variables evaluated in the present study in all the subjects, including controls (Table 5 and Figures 2A–D), indicated no correlation with dyspnea level at peak exercise (r = 0.0934), a weak correlation with peak *V'*_O2_ (r = −0.3219), slight correlation with *f*_R_ at peak exercise (which was the only ventilatory parameter) (r = 0.2122), weak correlation with O_2_-pulse at peak exercise as a cardiovascular parameter (r = −0.3331), good correlations with PaCO_2_ (r = −0.5250), and PetCO_2_ (r = −0.8542) as gas exchange parameters related to CO_2_ clearance, the strongest correlation with ΔFO_2_ (r = −0.8835) at peak exercise as a gas exchange parameter related to O_2_ extraction, and weak correlation with BMI (r = −0.2906). The correlations of the *V'*_E_/ *V'*_CO2_-nadir in all subjects were almost the same as those of the *V'*_E_-*V'*_CO2_-slope (Table 5). Only slight correlation was observed between the *V'*_E_/ *V'*_CO2_-intercept and the peak exercise ΔFO_2_ in all subjects including controls (r = 0.2155).

**Table 5:** Correlation of the parameters related to *V'*_E_-*V'*_CO2_-slope or *V'*_E_/*V'*_CO2_-nadir in all control subjects and COPD patients (*n* = 172).

	***V'***_*****E*****_ **-** ***V'***_**CO2**_ **-slope**		***V'***_*****E*****_ **/** ***V'***_**CO2**_ **-nadir**	
	***r***	***p*-value**	***r***	***p*-value**
**Peak incremental exercise parameters**				
Dyspnea, Borg scale	0.0934	0.2245	0.0520	0.4995
*V'*_O2_, mL · min^−1^ · kg	−0.3219	<0.0001	−0.5493	<0.0001
*V'*_E_, L · min^−1^	0.0479	0.5325	0.2178	0.0041
*V*_T_, mL	−0.0997	0.1932	−0.3467	<0.0001
*f*_R_, breaths · min^−1^	0.2122	0.0052	0.1210	0.1137
Ti/Ttot	−0.0384	0.6172	−0.2535	0.0008
*V'*_E_/ *V'*_O2_	0.8573	<0.0001	0.8446	<0.0001
*V*_D_/*V*_T_	0.3094	<0.0001	0.5769	<0.0001
HR, beats · min^−1^	−0.2201	0.0037	−0.3545	<0.0001
O_2_ pulse, mL · beats^−1^	−0.3331	<0.0001	−0.5615	<0.0001
ΔFO_2_, %	−0.8835	<0.0001	−0.9093	<0.0001
pH	0.4835	<0.0001	0.4441	<0.0001
PaO_2_, mmHg	−0.0686	0.3711	−0.1776	0.0198
PaCO_2_, mmHg	−0.5250	<0.0001	−0.3357	<0.0001
PetCO_2_, mmHg	−0.8542	<0.0001	−0.7819	<0.0001
HCO${}_{3}^{-}$, mEq· L^−1^	−0.3366	<0.0001	−0.1459	0.0561
Plasma lactate, mg · dL^−1^	−0.1959	0.0105	−0.3855	<0.0001
**Pulmonary function**				
FEV_1_, L	0.0419	0.5849	−0.1993	0.0088
FVC, L	0.1461	0.0559	−0.0564	0.4628
IC, L	−0.0214	0.7809	−0.1859	0.0146
**Others**				
Age, y	0.1498	0.0498	0.1988	0.0090
BMI, kg· m^−2^	−0.2906	0.0001	−0.3746	<0.0001

*ΔFO_2_, difference between inspired oxygen concentration and expired oxygen concentration; f_R_, breathing frequency; HR, heart rate; O_2_ pulse, V'_O2_/HR; PaCO_2_, arterial carbon dioxide tension; PaO_2_, arterial oxygen tension; PetCO_2_, partial pressure of end-tidal carbon dioxide, HCO${}_{3}^{-}$, bicarbonate ion; Ti/Ttot, inspiratory duty cycle; V'_CO2_, carbon dioxide output; V_D_/V_T_, physiologic dead space/tidal volume ratio; V'_E_, minute ventilation; V'_E_/V'_CO2_-nadir, the lowest value during exercise (see the Methods for details); V'_E_/ V'_CO2_-slope, the slope was determined by linear regression analysis of V'_E_ to V'_CO2_ obtained during exercise (see the Methods for details); V'_O2_, oxygen uptake; V_T_, tidal volume*.

### Correlations Between O_2_ Extraction and the Various Parameters

In all the study subjects including controls and in only COPD patients (Table 6), peak exercise ΔFO_2_ (1) correlated well with the gas exchange parameters related to CO_2_ clearance such as *V'*_E_/ *V'*_CO2_, *V*_D_/*V*_T_, PaCO_2_, and PetCO_2_ at peak exercise; (2) did not correlate with *V'*_E_ at peak exercise; (3) correlated more significantly with O_2_-pulse at peak exercise than with peak *V'*_O2_ or HR-*V'*_CO2_-slope; and (4) correlated positively with BMI. However, there was no correlation between BMI and peak *V'*_O2_ (r = 0.0896, *p* = 0.2425 for all subjects; r = 0.0981 *p* = 0.2232 for COPD patients).

**Table 6:** Correlations of the parameters related to the difference between inspired oxygen concentration and expired oxygen concentration (ΔFO_2_) in all the study subjects and COPD patients.

	**All subjects**		**COPD patients**	
	**(***n =*** 172)**		**(***n =*** 156)**	
	***r***	***p*-value**	***r***	***p*-value**
**Peak incremental exercise**				
Dyspnea, Borg scale	−0.0695	0.3677	−0.0611	0.4504
*V'*_O2_, mL · min^−1^ · kg	0.3728	<0.0001	0.2951	0.0002
*V'*_E_, L · min^−1^	0.0058	0.9397	−0.0991	0.2182
*V*_T_, mL	0.1665	0.0295	0.0974	0.2262
*f*_R_, breaths · min^−1^	−0.2179	0.0042	−0.2748	0.0005
Ti/Ttot	0.0795	0.3013	−0.0538	0.5046
*V'*_E_/ *V'*_CO2_	−0.9223	<0.0001	−0.9189	<0.0001
*V*_D_/*V*_T_	−0.3655	<0.0001	−0.3077	<0.0001
pH	−0.4062	<0.0001	−0.4202	<0.0001
PaO_2_, mmHg	0.0292	0.7043	−0.0777	0.3352
PaCO_2_, mmHg	0.4911	<0.0001	0.5681	<0.0001
PetCO_2_, mmHg	0.8514	<0.0001	0.8540	<0.0001
HCO${}_{3}^{-}$, mEq· L^−1^	0.3283	<0.0001	0.4078	<0.0001
Plasma lactate, mg · dL^−1^	0.1406	0.0682	0.0817	0.3140
HR, beats · min^−1^	0.1952	0.0105	0.1260	0.1172
O_2_ pulse, mL · beats^−1^	0.4543	<0.0001	0.4050	<0.0001
**During incremental exercise**				
HR-*V'*_CO2_-slope, beats · L^−1^	−0.1913	0.0122	−0.1400	0.0812
**Pulmonary function**				
FEV_1_, L	0.0275	0.7213	−0.1147	0.1538
FVC, L	−0.0490	0.5242	−0.1316	0.1014
IC, L	0.0772	0.3156	0.0195	0.8094
**Others**				
Age, years old	−0.1408	0.0662	−0.1243	0.1221
BMI, Kg· m^−2^	0.3871	<0.0001	0.4178	<0.0001

*ΔFO_2_, difference between inspired oxygen concentration and expired oxygen concentration; f_R_, breathing frequency; HR, heart rate; O_2_ pulse, V'_O2_/HR; PaCO_2_, arterial carbon dioxide tension; PaO_2_, arterial oxygen tension; PetCO_2_, partial pressure of end-tidal carbon dioxide, HCO${}_{3}^{-}$, bicarbonate ion; HR-V'_CO2_-slope, the slope was determined by using linear regression analysis of HR to V'_CO2_ obtained during the exercise (see the Methods for details); Ti/Ttot, inspiratory duty cycle; V'_CO2_, carbon dioxide output; V_D_/V_T_, physiologic dead space/tidal volume ratio; V'_E_, minute ventilation; V'_O2_, oxygen uptake; V_T_, tidal volume*.

## Discussion

All subjects across all four GOLD COPD stages and controls underwent CPET with an evaluation of exertional acidosis to investigate the cardiopulmonary response of ΔFO_2_ to exercise and the physiological implications of the *V'*_E_-*V'*_CO2_ relationship. The main findings of this study were as follows. First, as COPD progressed, (1) patients with more severe COPD had a slow and shallow respiratory pattern, because of the mechanical constraints on *V*_T_, which led to exertional respiratory acidosis, and greater dependence of their reduced peak *V'*_O2_ on peak exercise ΔFO_2_ due to the decreased dependence on *V'*_E_. In addition, the ratio of ΔFO_2_ to *V'*_E_ was higher with more severe COPD; and 2) although there was no significant difference in peak exercise ΔFO_2_ among the GOLD stages, the ΔFO_2_-*V'*_CO2_-slope became steeper and correlated well with the slope of the exertional respiratory acidosis level. Second, peak exercise ΔFO_2_ as a gas exchange parameter related to O_2_ extraction had the strongest correlation with ventilatory inefficiency related to CO_2_ clearance among the gas exchange parameters, mechanical ventilatory parameters, exercise tolerance and exertional cardiac function in all subjects including controls. Third, peak exercise ΔFO_2_ did not contribute significantly to *V'*_E_.

The excess or reduced ventilatory response to exercise based on the ventilatory capability in each cardiopulmonary disease might result from compensation for the pathophysiological condition that each impairment causes during exercise. In patients with HF or PAH, excess ventilation often occurs with a rapid shallow breathing pattern, to compensate for the exertional pathophysiological condition, which has been postulated to be due to (1) earlier onset of exertional lactic acidosis, (2) increased chemoreflex activity, (3) a restrictive lung mechanism, (4) reduction in alveolar-capillary gas diffusion, and (5) increased sympathetic activity (Sue, 2011). In humans, regardless of the underlying disease, pH-related homeostasis is commonly an important metabolic determinant of ventilatory control during exercise (Miki et al., 2009, 2012, 2013; Wasserman et al., 2014; Miki, 2021). Näveri et al. reported that the main limiting factor of exercise performance in both healthy subjects and HF patients is the development of lactic acidosis, although it occurs at different levels of exercise (Näveri et al., 1997). In the present study, in control subjects, exertional lactic acidosis occurred at peak exercise, followed by an adequate compensatory ventilatory response with tachypnea, which led to the low *V'*_E_-*V'*_CO2_-slope and *V'*_E_/*V'*_CO2_-nadir. In COPD, in contrast to PAH and HF, there is limited evidence of a relationship between ventilatory inefficiency and the ventilatory response to exercise. Neder et al. reported that the *V'*_E_-*V'*_CO2_-slope decreases with the increasing severity of COPD (Neder et al., 2015). In the present study as well, increasing levels of severity of COPD were not associated with a high degree of ventilatory inefficiency, and the slow and shallow breathing with a prolonged expiratory pattern led to exertional respiratory acidosis due to wasted ventilation (Table 2). Further, in the present study, in contrast to cardiovascular disease (Sue, 2011; Weatherald and Laveneziana, 2018), the ventilatory inefficiency correlated well with the gas exchange parameters related to exertional respiratory acidosis rather than with exertional lactic acidosis levels (Table 5). This could be explained by the previously reported result that the resultant hypercapnia (O'Donnell et al., 2002) at which carbon dioxide (CO_2_) is dominantly increased due to dynamic lung hyperinflation, led to a reduced *V'*_E_ for a given *V'*_CO2_, that is, low ventilatory inefficiency, in COPD. Interestingly, however, the ventilatory inefficiency related to CO_2_ clearance most strongly correlated with peak exercise ΔFO_2_, which is considered to represent the sum of body's O_2_ extraction among O_2_ delivery, O_2_ reservoirs, and O_2_ consumption from the body's reservoirs in all subjects in the present study (Figures 2A–D and Table 5). In addition, there was no significant difference in the change in ΔFO_2_ from rest to peak exercise or the change in *V'*_E_/*V'*_CO2_ from the value at rest to the nadir among all subjects in the present study (Table 2). Although little is so far known about the relationship between ΔFO_2_ and the ventilatory response to exercise, ventilatory efficiency might depend on optimal O_2_ extraction and CO_2_ clearance in the cardiopulmonary-muscle crosstalk during exercise.

O_2_ extraction evaluated by ΔFO_2_, which can be directly determined by non-invasive CPETs, correlated strongly with ventilatory inefficiency, and might contribute to compensating for the reduced exercise tolerance. The HR response to exercise in the present study showed that (1) HR at peak exercise and the quotient obtained by dividing the value by the predicted maximum HR decreased progressively, although the HR-*V'*_CO2_-slope increased from control to GOLD stage IV COPD (Table 2), that is, HR increased steeply during exercise because cardiac function decreased as the stage of COPD advanced, although the level of dysfunction was small; and (2) the peak exercise ΔFO_2_ correlated with O_2_-pulse (r = 0.4543), although it did not correlate well with the HR-*V'*_CO2_-slope, i.e., the degree of cardiac dysfunction in all subjects (Table 6). Given that the O_2_ pulse is equal to the product of stroke volume and the arteriovenous O_2_ difference, which depends on total O_2_ extraction from the body, and that ΔFO_2_ did not correlate well with the HR-*V'*_CO2_-slope or *V'*_E_ at peak exercise in the present study, peak exercise ΔFO_2_ might contribute to O_2_ extraction in the cardiopulmonary and peripheral muscle crosstalk rather than cardiac function or mechanical ventilation during exercise. In support of this, in the present study, a positive correlation between peak exercise ΔFO_2_ and BMI was confirmed (Table 6), although the relationship between ΔFO_2_ and muscle O_2_ extraction was not directly investigated in the present study. Interestingly, the ΔFO_2_-*V'*_CO2_-slope and the ratio of ΔFO_2_ to *V'*_E_ at peak exercise increased progressively as the stage of COPD advanced, although, unexpectedly, there was no significant intrastage difference in peak exercise ΔFO_2_ in the present study (Table 2). In addition, the ΔFO_2_-*V'*_CO2_-slope was positively correlated with the PaCO_2_-*V'*_CO2_-slope, that is, the degree of exertional respiratory acidosis (Table 4). These findings suggest that (1) patients with enough exercise tolerance did not necessarily need a high peak exercise ΔFO_2_, because such patients could depend on much of their ventilatory capability to increase oxygen uptake (Table 3) and (2) maintaining cardiopulmonary and peripheral muscle crosstalk is helpful to compensate for O_2_ demand, especially in patients with advanced COPD who predominantly have ventilatory impairments with exertional respiratory acidosis, because of the relatively high dependence of their reduced peak *V'*_O2_ on peak exercise ΔFO_2_ compared with its dependence on *V'*_E_ in GOLD IV cases (Table 3). This compensatory or protective mechanism of ΔFO_2_ was not clearly proven in the present study. However, increasing the ΔFO_2_ level or the slope of ΔFO_2_ during exercise might be a therapeutic target for improving ventilatory inefficiency or exercise tolerance in COPD patients, this target being independent of the target of improving ventilatory capability (Figures 2B,D). Therefore, focusing on the two factors related to *V'*_O2_, that is, ΔFO_2_ and *V'*_E_ may be useful for developing therapeutic strategies to increase exercise tolerance. This is likely supported by the therapeutic variation pattern based on the two factors related to *V'*_O2_, that is, the arteriovenous O_2_ difference and cardiac output that Cattadori et al. reported graphically to evaluate the effect of exercise training in HF patients (Cattadori et al., 2011). In addition, increased exertional muscle acidosis was associated with the down-regulation of skeletal muscle oxidative enzyme activity (Kutsuzawa et al., 1992). Recent evidence by Bruce et al. suggests that abnormal peripheral muscle metaboreflexes are responsible for the excess ventilation in COPD patients (Bruce et al., 2016). In cardiopulmonary diseases, identification of not only cardiopulmonary responses but also the peripheral muscle condition is commonly useful to facilitate personalized care based on each individual's dysfunction (Shelton et al., 2010; Vogiatzis and Zakynthinos, 2013; Maekura et al., 2015; Miki, 2021). In advanced COPD, measurement of ΔFO_2_ might be informative for identifying ventilatory inefficiency, and might be a compensatory component for O_2_ availability in the body's cardiopulmonary and peripheral muscle crosstalk.

This study has some limitations. First, selection bias due to this being a single-center study, lack of data on pulmonary hypertension, and the low percentage of COPD patients regularly taking long-acting β_2_-agonists and/or long-acting muscarinic antagonists might have affected the results. Second, to investigate the direct relationship between ΔFO_2_ and muscle O_2_ extraction, the evaluation of peripheral exertional tissue oxygenation (Chuang et al., 2019), which was not performed in the present study might be more informative. Third, in cardiovascular disease, investigating of the relationship between ventilatory inefficiency and ΔFO_2_ is necessary to confirm the increased appreciation of not only excess ventilation but also reduced O_2_ extraction as a marker of greater disease severity or worse outcomes. Given the report of Chuang et al. (2019), the conflicting evidence that excess ventilation is seen with the progression of cardiac disease, but not with the progression of COPD, might be related to the fact that exertional muscle oxygenation is more impaired in cardiac disease than in COPD as a pathophysiological mechanism of ventilatory inefficiency. Fourth, because many parameters in CPET are calculated using the basic parameters of gas flow, O_2_ concentration and CO_2_ concentration, multicollinearity due to variable selection should be evaluated. However, ΔFO_2_ was directly determined as an actual value and was not used in the calculation of ventilatory inefficiency, with which it had the strongest correlation.

## Conclusion

Ventilatory efficiency might depend on the exertional change in O_2_ extraction and CO_2_ clearance in the body. Measuring ΔFO_2_ could be a key component for identifying ventilatory inefficiency related to CO_2_ clearance and O_2_ availability in the cardiopulmonary and peripheral muscle crosstalk of COPD. Individualized care to increase ΔFO_2_ based on the specific dysfunction in COPD would help to improve both exercise tolerance and ventilatory inefficiency.

## Data Availability Statement

The raw data supporting the conclusions of this article will be made available by the authors, without undue reservation.

## Ethics Statement

The studies involving human participants were reviewed and approved by the institutional review board of the National Hospital Organization Osaka Toneyama Medical Center. The patients/participants provided their written informed consent to participate in this study.

## Author Contributions

KM was responsible for the study conception and design. KM, KT, RM, TM, MM, HH, HK, TKa, TKu, and HK were responsible for data acquisition, analysis, interpretation, were responsible for drafting, and revising the article. Each author approved the submission of this manuscript for publication. All authors contributed substantially to this article.

## Conflict of Interest

The authors declare that the research was conducted in the absence of any commercial or financial relationships that could be construed as a potential conflict of interest.

## Publisher's Note

All claims expressed in this article are solely those of the authors and do not necessarily represent those of their affiliated organizations, or those of the publisher, the editors and the reviewers. Any product that may be evaluated in this article, or claim that may be made by its manufacturer, is not guaranteed or endorsed by the publisher.

## Footnotes

^1^2020 Global Strategy for Prevention, Diagnosis, and Management of COPD. Global Initiative for Chronic Obstructive Lung Disease. Available online at: https://goldcopd.org/gold-reports/ (accessed December 20, 2020).

## Abbreviations

CPET: cardiopulmonary exercise testing
ΔFO_2_: difference between inspiratory and expiratory oxygen concentrations
GOLD: Global Initiative for Chronic Obstructive Lung Disease
ICS: inhaled corticosteroid
LABA: long-acting β2-agonist
LAMA: long-acting muscarinic antagonist
SABA: short-acting β2-agonist
SAMA: short-acting muscarinic antagonist.
