# Corrigendum: Oxygen Extraction Based on Inspiratory and Expiratory Gas Analysis Identifies Ventilatory Inefficiency in Chronic Obstructive Pulmonary Disease

**DOI:** 10.3389/fphys.2021.770151

**Published:** 2021-09-30

**Authors:** Keisuke Miki, Kazuyuki Tsujino, Ryoji Maekura, Takanori Matsuki, Mari Miki, Hisako Hashimoto, Hiroyuki Kagawa, Takahiro Kawasaki, Tomoki Kuge, Hiroshi Kida

**Affiliations:** ^1^Department of Respiratory Medicine, National Hospital Organization Osaka Toneyama Medical Center, Toyonaka, Japan; ^2^Graduate School of Health Care Sciences, Jikei Institute, Osaka, Japan

**Keywords:** acidosis, carbon dioxide, cardiac function, exercise, gas exchange, ventilatory efficiency

The author's name was incorrectly spelt as Ryoji Maekuara. The correct spelling is Ryoji Maekura.

The author's name was incorrectly spelt as “RyojiMaekuara”. It should be spelt as “Ryoji Maekura.”

In the citation, the author's name was incorrectly listed as “Maekuara R”. It should be listed as “Maekura R.”

In the copyright statement, the author's name was incorrectly spelt as “Maekuara”. It should be spelt as “Maekura.”

The authors apologize for this error and state that this does not change the scientific conclusions of the article in any way. The original article has been updated.

## Publisher's Note

All claims expressed in this article are solely those of the authors and do not necessarily represent those of their affiliated organizations, or those of the publisher, the editors and the reviewers. Any product that may be evaluated in this article, or claim that may be made by its manufacturer, is not guaranteed or endorsed by the publisher.

